# Anti-obesity effects of Yerba Mate (*Ilex Paraguariensis*): a randomized, double-blind, placebo-controlled clinical trial

**DOI:** 10.1186/s12906-015-0859-1

**Published:** 2015-09-25

**Authors:** Sun-Young Kim, Mi-Ra Oh, Min-Gul Kim, Han-Jeoung Chae, Soo-Wan Chae

**Affiliations:** Departments of Medical Nutrition Therapy, Chonbuk National University Medical School, 567 Baekje-daero, Deokjin-gu, Jeonju, Jeonbuk 561-756 Republic of Korea; Clinical Trial Center for Functional Foods, Chonbuk National University Hospital, 20 Geonji-ro, Jeonju, Jeonbuk 561-712 Republic of Korea; Clinical Trial Center, Chonbuk National University Hospital, 20 Geonji-ro, Jeonju, Jeonbuk 561-712 Republic of Korea; Department of Pharmacology, Chonbuk National University Medical School, 567 Baekje-daero, Deokjin-gu, Jeonju, Jeonbuk 561-756 Republic of Korea

**Keywords:** Yerba Mate, *Ilex paraguariensis*, Obesity, Clinical trials

## Abstract

**Background:**

Obesity is a major health problem. A food field research that has recently aroused considerable interest is the potential of natural products to counteract obesity. Yerba Mate may be helpful in reducing body weight and fat. The aim of this study was to investigate the efficacy and safety of Yerba Mate supplementation in Korean subjects with obesity.

**Methods:**

A randomized, double-blind, placebo-controlled trial was conducted. Subjects with obesity (body mass index (BMI) ≥ 25 but < 35 kg/m^2^ and waist-hip ratio (WHR) ≥ 0.90 for men and ≥ 0.85 for women) were given oral supplements of Yerba Mate capsules (*n* = 15) or placebos (*n* = 15) for 12 weeks. Subjects take three capsules per each meal, total three times in a day (3 g/day). Measured outcomes were efficacy (abdominal fat distribution, anthropometric parameters and blood lipid profiles) and safety (adverse events, laboratory test results and vital signs).

**Results:**

During 12 weeks of Yerba Mate supplementation, decreases in body fat mass (*P* = 0.036) and percent body fat (*P* = 0.030) compared to the placebo group were statistically significant. WHR was significantly decreased (*P* = 0.004) in the Yerba Mate group compared to the placebo group. No clinically significant changes in any safety parameters were observed.

**Conclusions:**

Yerba Mate supplementation decreased body fat mass, percent body fat and WHR. Yerba Mate was a potent anti-obesity reagent that did not produce significant adverse effects. These results suggested that Yerba Mate supplementation may be effective for treating obese individuals.

**Trial registration:**

ClinicalTrials.gov: (NCT01778257)

**Electronic supplementary material:**

The online version of this article (doi:10.1186/s12906-015-0859-1) contains supplementary material, which is available to authorized users.

## Background

Obesity is a major health problem. Overweight and obesity are defined by the World Health Organization (WHO) as abnormal or excessive fat accumulation that increases the risk of type 2 diabetes, cardiovascular disease and several types of cancers [1, 2]. Obesity continues to be one of the biggest global challenges in the 21st century, with at least 2.8 million adults dying each year from conditions resulting from being overweight and obeses [3].

Many efforts to overcome obesity have focused on developing anti-obesity agents. Among drugs, fenfluramine, dexfenfluramine and sibutramine reduce energy intake, suppress hunger and enhance satiety [4]. However, only a few of these drugs enter and stay in the market because most are associated with serious side effects. Common side effects include insomnia, dry mouth, constipation, nausea and headache. In addition, sibutramine is associated with increased blood pressure and pulse rate, ventricular and supraventricular tachyarrhythmias and angina pectoris, raising concerns about a potential increase in cardiac risk [5, 6]. Because of potential adverse side effects, anti-obesity drugs are recommended to be prescribed for obesity only when the benefits of treatment clearly outweigh the risks. Whereas drug for antiobesity is effective but with some concern about side effect, most functional foods that are generally considered safe have not been scientifically validated for treating obesity or their effects are not statistically significant [7, 8].

Yerba Mate, the dried leaves of the plant *Ilex paraguariensis*, is currently consumed by over 1 million people worldwide, traditionally in many South American countries including Argentina, Brazil, Uruguay, Paraguay. More recently, Yerba Mate tea has been consumed in North America and Europe [9]. Yerba Mate beverages are reported to have biological activities, probably due to their high polyphenol content. Phenolic compounds have long been known to possess biological functions. In addition to polyphenols such as flavonoids (quercetin and rutin) and phenolic acids (chlorogenic and caffeic acids), Yerba Mate is also rich in caffeine and saponins [10]. Yerba Mate extracts are especially rich in chlorogenic acids that might contribute to hypocholesterolemic [11] and weight loss effects [12]. Chlorogenic acid inhibits adipogenesis by reducing the expression of genes regulating adipogenesis in 3T3-L1 cells and in mouse model of HFD-induced obesity [13]. In these regards, it is likely that Yerba Mate may potential alternative for controlling body fat accumulation and weight. *In vitro* and *in vivo* studies have demonstrated that Yerba Mate modulates signaling pathways, has chemopreventive activities [14, 15], enhance intestinal propulsion [16], has vasodilatation effects [17], inhibits glycation [18], inhibits oxidative stress [19] and has inflammatory effects [20]. Yerba Mate suppresses body weight gain and visceral fat accumulation and decreases serum levels of cholesterol, triglycerides, LDL cholesterol [21, 22]. Our previous study reported that Yerba Mate reduces body weight in mice with obesity induced by a high-fat diet. After feeding animals Yerba Mate for 4 weeks, we observed a decrease in total cholesterol, leptin levels and blood glucose that ultimately led to reduce their body weight [23]. Taking into account that Yerba Mate is especially rich in chlorogenic acid and several bioactive compounds, it suggest that it is possible to inhibit obesity.

Based on encouraging results from animal studies, we performed a clinical trial to evaluate the anti-obesity effects of Yerba Mate on humans. The objective of the study was to document the effects of 12 weeks of Yerba Mate supplementation on body fat composition in obese Korean people using a randomized, double-blind, placebo-controlled protocol.

## Methods

### Study design

The current study was conducted under the 12 weeks, randomized, double-blind, placebo-controlled clinical trial according to a computer-generated randomization schedule designed to assign subjects to Yerba Mate or placebo groups. Neither the investigators nor the subjects knew the randomization code or the results of the blood parameter until after statistical analysis was complete. Subjects attended a screening visit at which inclusion and exclusion criteria were assessed. Screening visit evaluation, physical examination and blood parameter screening tests were conducted for all subjects within 4 weeks of initial screening. A random number between 1 and 30 was generated for each participant and enrollees were scheduled for a first visit and randomly assigned to either the Yerba Mate (*n* = 15) or placebo (*n* = 15) group. Yerba Mate and placebo capsules were given to subjects every 6 weeks.

During the 12 weeks intervention period, subjects were asked to continue their usual diet but not other functional foods or dietary supplements. Computed tomography (CT) imaging and testing of biochemical parameters were evaluated before and after the intervention period for both groups. Every 6 weeks anthropometric measurements, lipid profile, vital signs and nutrient intake, adverse events, lifestyle and capsule compliance were evaluated. During the run-in phase of the trial, all subjects were instructed to maintain their normal diet and physical activity. A CONSORT checklist for the reporting of this study can be found in Additional file 1.

### Subjects

From July 2011 through May 2012, subjects were recruited at the Clinical Trial Center for Functional Foods (CTCF2) of Chonbuk National University Hospital (Jeonju, Republic of Korea). A total of 37 volunteers agreed to participate. Only individuals who were obese (body mass index (BMI) < 35 and ≥ 25 kg/m^2^ and waist-hip ratio (WHR) ≥ 0.90 for men or ≥ 0.85 for women) according to the Asia-Pacific guidelines and were not diagnosed with any other diseases were included.

The 30 subjects who met the study criteria (age, 43.2 ± 10.6 years; weight, 71.56 ± 10.14 kg; BMI 27.98 ± 2.68 kg/m^2^) were randomly divided into two groups (*n* = 15 each) and given either Yerba Mate (3150 mg/day) or a placebo (3150 mg/day). Exclusion criteria were: (a) significant variation in weight (more 10 %) in the past 3 months; (b) history of cardiovascular disease, gastrointestinal disease (Crohn’s disease), previous surgery (caesum or enterocele surgery), renal disease or abnormal hepatic function; (c) participation in another clinical trial within the past 3 months; (d) antipsychotic drug therapy within past the 2 months; (e) pregnancy or breast feeding; (f) history of alcohol or substance abuse; or (g) allergies or hypersensitivity to any of the ingredients in the test products. All subjects provided written informed consent before the investigation commenced. Study protocols were approved by the Institutional Review Board of Chonbuk National University Hospital (IRB number: CUH 2011–07–18). The protocol is registered at www.clinicaltrials.gov (NCT01778257).

### Test supplement

Yerba Mate was a standardized product containing chlorogenic acid 35 mg/g and was prepared by JEJU TECHNOPARK (Jeju, Republic of Korea). Dried Yerba Mate leaves were collected from Argentina and extracted with water at 100 °C for 2 h. Extracts were filtered and concentrated under reduced pressure to 20 brix at 40–50 °C, dried at 160–180 °C using a spray dryer. Yerba Mate was administered in capsule form (333.38 mg Yerba Mate and 16.6 mg diluting agents in a 350 mg capsule). Administration was 3 g/day Yerba Mate water extract, the recommended daily dose from Korea Food & Drug Administration (KFDA) in our clinical trial. The appearance of the Yerba Mate and placebo capsules was identical (Additional file 2).

Subjects take three capsules per each meal, total three times in a day (before breakfast, lunch and dinner). Yerba Mate and placebo capsule packaging was indistinguishable and was labeled with the subject number. Subjects were instructed to bring all remaining supplements to each visit and were withdrawn if supplement consumption was < 70 % of the recommended dose.

### Efficacy outcome measurements

The 30 subjects who met the study criteria were asked to visit the clinic once every 6 weeks (weeks 0, 6 and 12 of the study period) for four clinical visits including the initial screening. During each visit, current supplementation use was reviewed and symptoms or side effects were recorded. During the screening visit, demographic and lifestyle information was collected (gender, age, alcohol consumption and smoking). A medical history was taken and a urine pregnancy test was conducted.

The following parameters were assessed: abdominal fat distribution was measured and analyzed using CT (Somatom Sensation 16 MDCT; Siemens, Forchheim, Germany) before (0 week) and after the 12 weeks intervention period. Body weight, BMI, body fat mass, percent body fat and lean body mass were measured using the Inbody 3.0 (Biospace, Seoul, Korea) during each visit. Blood samples were collected after a minimum 12-h fast during initial screening and at the 6 weeks and 12 weeks intervention period to obtain lipid profiles changes of total cholesterol, triglycerides, high-density lipoprotein (HDL), low-density lipoprotein (LDL), and free fatty acids. Blood samples were analyzed with a Hitachi 7600–110 analyzer (Hitachi High Technologies, Tokyo, Japan) using standard methods in the biochemical laboratory of Chonbuk National University Hospital.

### Safety and dietary evaluation

Extract safety was assessed by the following procedures: hematology and laboratory tests were conducted during screening, 0 week and 12 weeks intervention periods for white blood cell (WBC), red blood cell (RBC), platelet counts, hemoglobin, hematocrit, total protein, albumin, alanine transaminase (ALT), aspartate transaminase (AST), blood urea nitrogen (BUN) and creatinine levels. Pulse and blood pressure were measured at every visit after a 5-min rest using an IntelliVue MP70 (Philips, Eindhovan, Netherlands). A personal report was also recorded. Subjects maintained their usual diet and activity and all completed a dietary record at each visit during the intervention period to evaluate energy intake and diet quality. Dietary intake data were analyzed by the same dietitian using CAN-pro 3.0 software (The Korea Nutrition Society, Seoul, Korea).

### Statistical analysis

Statistical analyses were performed using SAS software, version 9.2 (SAS Institute, USA). Data are presented as mean ± standard deviation (SD). The appropriate sample size was statistically determined to obtain a power of 80 % with an alpha of 0.05. This study was planned as a feasibility study, and the minimum sample size needed to detect. In order to demonstrate decrease of body fat mass, a sample size of 12 was required under the assumption that decrease of body fat mass is 3 kg with a standard deviation of 2.6 kg. Assuming a 20 % loss to follow-up, we set the total sample size at 30. Intention-to-treat (ITT) analysis included all randomized subjects who received at least one dose of Yerba Mate or placebo capsule. Efficacy and safety parameters of the ITT group were analyzed. For statistics we used a mixed effect model approach for ITT analysis with missing values [24]. General characteristics were analyzed by independent *t*-test or Chi-square tests. The significance of the differences within or between groups was tested by repeated measures ANOVA and a paired *t*-test of the mean. Chi-square tests were performed to determine differences in the frequencies of categorized variables between groups. A *P* value <0.05 was considered statistically significant.

## Results

### Subjects

**Fig. 1:**
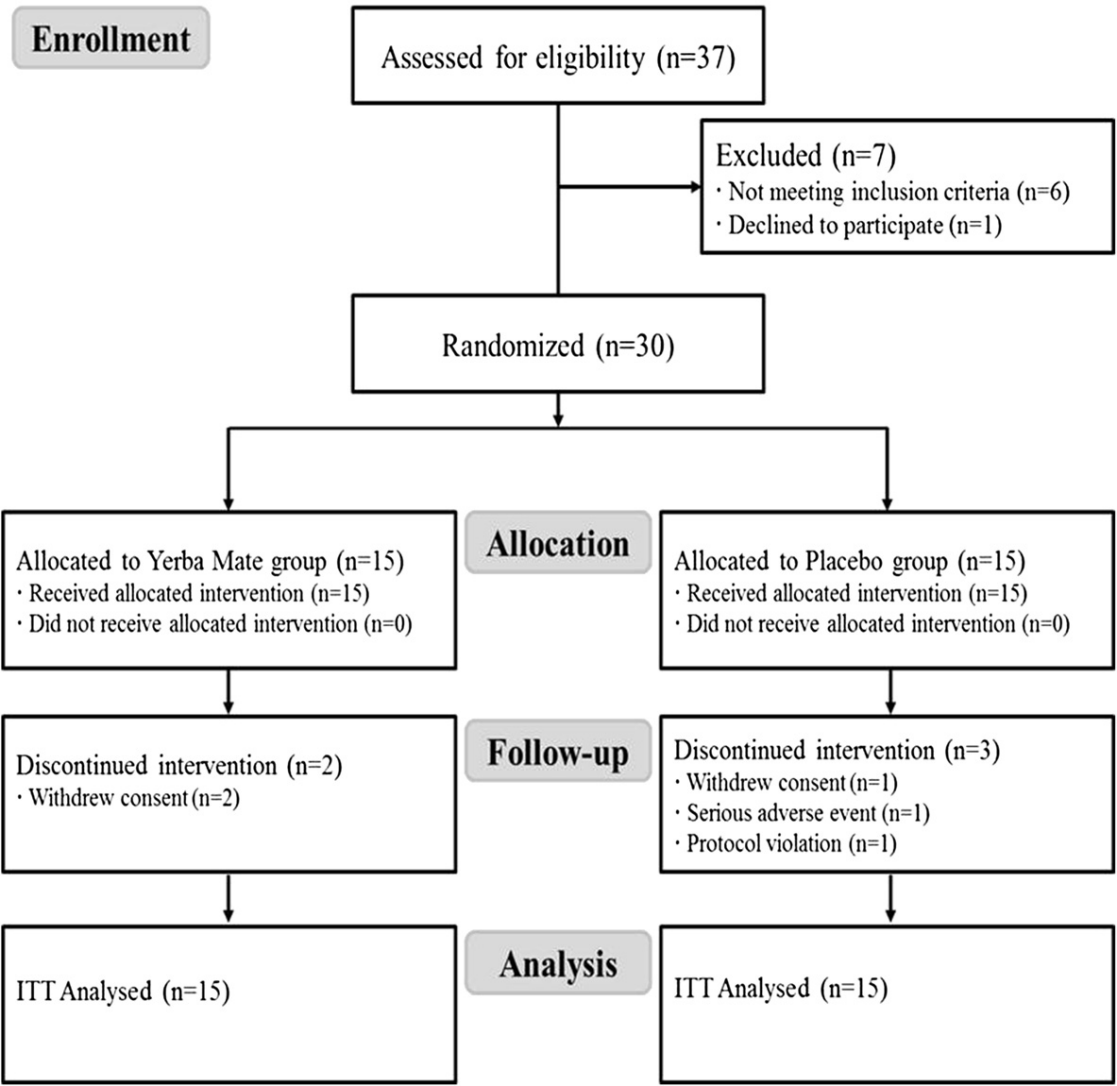
CONSORT diagram showing participant flow through the 12 weeks intervention. ITT, intention-to-treat population

Among the 37 subjects screened, 7 were excluded due to anthropometric characteristic or laboratory test result criteria. The remaining 30 fulfilled the inclusion criteria and were divided equally into Yerba Mate and placebo groups. Three withdrew consent for personal reasons, one discontinued treatment because of adverse side effects and one had a protocol violation (inclusion/exclusion criteria violation). Thus, 25 subjects (13 Yerba Mate and 12 placebo group members) finished the study (Fig. 1).

### Subject characteristics

General subject characteristics are in Table 1. No significant difference was observed in baseline characteristics such as age, height, weight and BMI between the Yerba Mate and placebo groups.

**Table 1:** Demographic characteristics of the study subjects

	Yerba Mate (*n* = 15)	Placebo (*n* = 15)	Total (*n* = 30)	*P* value^1)^
Age (year)	41.5 ± 11.6	44.9 ± 9.6	43.2 ± 10.6	0.379
Height (cm)	160.93 ± 6.95	158.40 ± 7.59	159.67 ± 7.27	0.349
Weight (kg)	74.50 ± 9.83	68.61 ± 9.89	71.56 ± 10.14	0.113
BMI (kg/m^2^)	28.65 ± 2.09	27.31 ± 3.10	27.98 ± 2.68	0.174
Sex				
Male^2)^	2 (13.3)	2 (13.3)	4 (13.3)	
Female	13 (86.7)	13 (86.7)	26 (86.7)	>0.999^3)^
Drinker				
Yes	8 (53.3)	6 (40)	14 (46.7)	
No	7 (46.7)	9 (60)	16 (53.3)	0.464
Smoker				
Yes	0 (0.0)	2 (13.3)	2 (6.7)	
No	15 (100)	13 (86.7)	28 (93.3)	0.483

Values are presented as the mean ± S.D

^1)^ Analyzed by independent *t* test

^2)^ *N* (%)

^3)^ Analyzed by chi-square tests

### Dietary assessment

No significant differences in dietary intake (calorie, carbohydrate, protein and fat) were observed between the groups during the intervention period (Additional file 3).

### Body fat composition

**Fig. 2:**
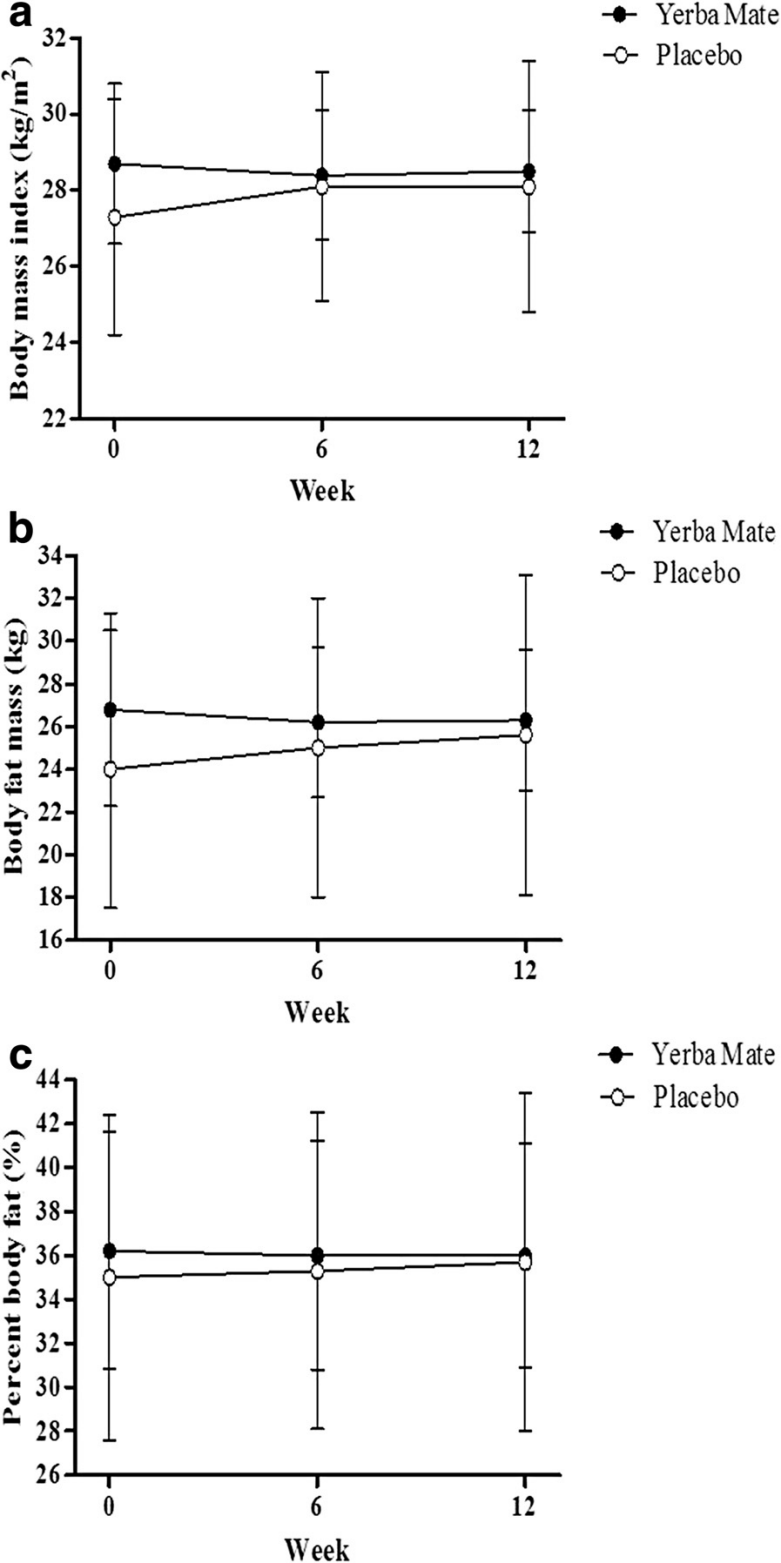
Effects on body fat composition. **a** Change in BMI during the study period. **b** Change in body fat mass during the study period. The Yerba Mate group was different when compared to placebo group (*P* = 0.036). **c** Change in percent body fat during the study period. The Yerba Mate group was different compared to placebo group (*P* = 0.030). Values are presented mean ± S.D for 15 subjects. Analyzed by repeated measures ANOVA and the *P* value represents the comparison to the placebo group (*P* < 0.05)

Changes in BMI, body fat mass and percent body fat at 0, 6 and 12 weeks of intervention are in Fig. 2. The decreased in body fat mass (*P* = 0.036) and percent body fat (*P* = 0.030) in the Yerba Mate group compared to the placebo group was significant.

### Abdominal fat

Changes in total abdominal fat, abdominal visceral fat and abdominal subcutaneous fat before and after the 12 weeks intervention period were analyzed (Table 2). After 12 weeks, no significant differences were found in area of total abdominal fat. Areas of abdominal visceral and subcutaneous fat tended to decrease in the Yerba Mate group, although no significant differences were observed between the two groups.

**Table 2:** Abdominal fat area of the Yerba Mate and placebo groups measured at 0 and 12 weeks

	Yerba Mate (*n* = 15)			Placebo (*n* = 15)			*P* value^2)^
	0 weeks	12 weeks	*P* value^1)^	0 weeks	12 weeks	*P* value^1)^	
Visceral fat (cm^3^)	1106.8 ± 387.5	1001.9 ± 338.4	0.145	947.0 ± 256.8	957.8 ± 353.7	0.695	0.181
Subcutaneous fat (cm^3^)	3123.5 ± 736.2	3101.2 ± 590.4	0.259	2984.0 ± 1040.7	3103.3 ± 1282.3	0.333	0.899
Visceral subcutaneous ratio	0.4 ± 0.1	0.3 ± 0.1	0.140	0.3 ± 0.1	0.3 ± 0.2	0.861	0.175

Values are presented as the mean ± S.D

^1)^ Analyzed by paired *t* test

^2)^ Analyzed by repeated measures ANOVA

### Lipid profile changes

Changes in serum level of total cholesterol, triglycerides, LDL cholesterol, HDL cholesterol and free fatty acids at 0, 6 and 12 weeks of intervention are in Table 3. At 12 weeks, free fatty acids levels tended to decrease and HDL cholesterol levels tended to increase in the Yerba Mate group. However, no significant differences were found between the Yerba Mate and placebo groups.

**Table 3:** Lipid profile changes of the Yerba Mate and placebo groups at 0, 6 and 12 weeks

	Yerba Mate (*n* = 15)				Placebo (*n* = 15)				*P* value^1)^
	0 weeks	6 weeks	12 weeks	*P* value^1)^	0 weeks	6 weeks	12 weeks	*P* value^1)^	
Total cholesterol (mg/dL)	193.6 ± 24.8	197.9 ± 33.2	204.4 ± 36.0	0.384	176.7 ± 28.8	172.3 ± 33.9	174.0 ± 28.4	0.977	0.722
HDL-cholesterol (mg/dL)	45.4 ± 7.7	48.0 ± 10.3	48.2 ± 10.1	0.424	52.3 ± 12.0	50.4 ± 8.3	48.0 ± 8.3	0.369	0.223
LDL-cholesterol (mg/dL)	120.9 ± 27.5	117.8 ± 28.8	121.7 ± 34.6	0.755	101.7 ± 25.6	97.2 ± 27.5	96.7 ± 30.8	0.944	0.862
Triglyceride (mg/dL)	141.5 ± 79.9	130.6 ± 96.3	154.2 ± 96.6	0.325	123.1 ± 65.8	107.2 ± 53.8	127.5 ± 95.1	0.548	0.969
Free Fatty Acid (μEq/L)	516.6 ± 163.6	412.2 ± 128.8	443.5 ± 169.7	0.234	541.5 ± 337.3	541.5 ± 225.6	575.3 ± 220.1	0.286	0.224

Values are presented as the mean ± S.D

^1)^ Analyzed by repeated measures ANOVA

### Anthropometric parameters

Changes in anthropometric parameters (height, weight, waist circumference, hip circumference, WHR, arm circumference and thigh circumference) at 0, 6 and 12 weeks of intervention are presented in Table 4. Decreased WHR (*P* = 0.005) in the Yerba Mate group compared to the placebo group were significant.

**Table 4:** Anthropometric parameters of the Yerba Mate and placebo groups at 0, 6 and 12 weeks

	Yerba Mate (*n* = 15)				Placebo (*n* = 15)				*P* value^1)^
	0 weeks	6 weeks	12 weeks	*P* value ^1)^	0 weeks	6 weeks	12 weeks	*P* value^1)^	
Height (cm)	160.9 ± 7.0	160.6 ± 7.1	160.6 ± 7.1	-	158.4 ± 7.6	158.5 ± 8.2	159.3 ± 8.0	-	-
Weight (kg)	74.5 ± 9.8	73.5 ± 9.2	73.8 ± 9.0	0.742	68.6 ± 9.9	70.8 ± 10.1	71.4 ± 9.5	0.472	0.387
Waist circumference (cm)	92.9 ± 6.2	90.8 ± 4.9	91.3 ± 5.1	0.170	90.1 ± 7.3	91.1 ± 7.8	91.9 ± 8.0	0.529	0.113
Hip circumference (cm)	100.1 ± 4.4	100.2 ± 4.0	100.9 ± 4.4	0.086	97.8 ± 5.0	98.5 ± 5.1	99.0 ± 5.3	0.971	0.478
Waist-hip ratio	0.93 ± 0.05	0.91 ± 0.04^†^	0.91 ± 0.04^‡^	0.003	0.92 ± 0.04	0.92 ± 0.04	0.93 ± 0.04	0.511	0.005
Arm circumference (cm)	33.4 ± 2.4	33.0 ± 2.3	33.0 ± 2.0	0.394	31.8 ± 2.4	32.3 ± 3.2	32.5 ± 2.6	0.869	0.489
Thigh circumference (cm)	53.8 ± 4.2	53.5 ± 4.5	53.5 ± 4.5	0.900	51.2 ± 3.8	52.0 ± 3.9	52.3 ± 3.9	0.535	0.495

Values are presented as the mean ± S.D

^1)^ Analyzed by repeated measures ANOVA. Statistically significant compared to the placebo group

^†^ 0 vs. 6 weeks: waist-hip ratio, *P* = 0.004

^‡^ 0 vs. 12 weeks: waist-hip ratio, *P* = 0.002

### Safety analyses

Results of subjects’ complete blood cell count, liver and kidney function tests and vital signs were within normal ranges for both the Yerba Mate and placebo groups throughout the study. Suggesting that Yerba Mate supplementation did not cause any adverse side effects (Additional files 4, 5 and 6).

## Discussion

Our randomized, placebo-controlled, double-blind clinical trial evaluated the safety and efficacy of Yerba Mate as a supplement for obese Korean people. After 12 weeks of supplementation, we observed a significant decreased in body fat mass and percent body fat. WHR also decreased significantly in the Yerba Mate group compared to the placebo group. Unlike currently prescribed anti-obesity drugs, subjects did not report any specific adverse events.

Our study showed decreased body fat in subjects taking a Yerba Mate-only supplement. Despite evidence from previous research supporting the weight-reducing potential of Yerba Mate, this is the first controlled trial ascribing an anti-obesity effect to the ingestion of this herb. Yerba Mate was previously tested in a supplement with other ingredients such as green tea, asparagus, black tea, guarana and kidney bean extracts [25]. Another clinical trial of 6 weeks of treatment with green mate powder extract (1200 mg/day) showed significant decreased in body fat mass and percent body fat [26]. In our clinical trial, subjects who completed 12 weeks of treatment with Yerba Mate showed decreased WHR, body fat mass, percent body fat and trends in decreased visceral fat and visceral/subcutaneous fat. Our previous animal study showing that adipocyte size decreased in a group given Yerba Mate [23]. Yerba Mate decreases the differentiation of preadipocytes and reduces accumulation of lipids in adipocytes [27]. These results indicate that decreasing adipose tissue growth, body fat mass (kg), percent body fat (%) and obesity of Yerba Mate. The potential thermogenic properties of significantly increasing resting energy expenditure (REE) for at least four hours post-ingestion in moderated-level habitual caffeine consumers [28]. The increase in REE might have been due to a 340-mg proprietary blend of caffeine anhydrous, guarana, yerba mate and green tea extract. Another study evaluated the acute effects of oral administration of 12 commercially available plant preparations aimed at treating human obesity [29]. Only treatment with a green mate extract changed the respiratory quotient, indicating a rise in the proportion of oxidized fat that might have led to decreased body fat. These results suggest the potential of mate leaves for treatment of obesity.

In our study, a decrease in free fatty acid was observed with Yerba Mate, although the difference from the placebo group was not significant. Mate tea also affects other aspects of lipid metabolism. *I. paraguariensis* extract inhibits atherosclerosis progression in cholesterol-fed rabbits, although it does not decrease serum cholesterol [22]. A decrease in serum cholesterol and triacylglycerol concentrations was observed after ingestion of Yerba Mate extracts in rats fed a high-cholesterol diet [30]. A previous animal study showed that total cholesterol decreased in a group given Yerba Mate [23]. Previous studies assumed that antioxidants in *I. paraguariensis* A. St.-Hil extracts inhibit LDL oxidation *in vitro* and *in vivo*, with a potency comparable to ascorbic acid [31, 32]. Another clinical trial observed a decreased in serum concentration of cholesterol and triacylglycerols [26]. Decreased LDL-cholesterol was observed in normolipidemia and dyslipidemia [33]. Whether metabolic benefits from weight decrease occur after long-term treatment with Yerba Mate products remains to be determined.

Yerba Mate leaf extracts contain components such as chlorogenic acid (monocaffeoylquinic and dicaffeoylquinic acids), hydroxycinnamic acids (caffeic acid, quinic acid) and numerous triterpenic saponins [34]. Chlorogenic acid is one of the most abundant polyphenols in fruits such as plums, apples and cherries and has been shown to reduce body weight as well as improve lipid metabolism and levels of obesity-related hormones in mice [12]. Chlorogenic acid inhibits adipogenesis by reducing the expression of genes regulating adipogenesis in 3T3-L1 cells and in mouse model of HFD-induced obesity [13]. The mechanisms of Yerba Mate on fat decrease, while not directly known, might be due to effects of single components, as investigated in previous studies. The Yerba Mate used in our study contained 35 mg/g chlorogenic acid. Yerba Mate extracts are especially rich in chlorogenic acids that might contribute to hypocholesterolemic [11] and weight loss effects [12]. Our previous animal study showing that adipocyte size decreased in a group given Yerba Mate [23] and decreases the differentiation of preadipocytes and reduces accumulation of lipids in adipocytes [27]. Additionally, Yerba Mate inhibits body weight gain and visceral fat accumulation and decreases serum levels of cholesterol, triglycerides, LDL cholesterol [21, 22]. In these regards, it is likely that Yerba Mate may potential alternative for controlling body fat accumulation and weight.

Our study has a limited ability to draw broad conclusions. First, there is much controversy concerning whether smokers are actually thinner than nonsmokers. However, nicotine may be an appetite suppressant and influence an individual’s eating habits [35]. Although smokers are included in the placebo group of this study, the results were not affected regardless of inclusion smokers and therefore further study will be designed to consider this point. Second, another study reported that Yerba Mate preparation might reduce energy intake and increase satiety, while inducing weight loss. A weight loss effect was not demonstrated definitively, although weight and abdominal fat diminished more in the Yerba Mate group than in the placebo group. This result might be due to the low statistical power of our study because of the limited sample size. Moreover, small sample size in this study limits the generalization of our results to other populations with obesity. Our study has an exploratory clinical trial using standardized Yerba Mate and this study is insufficient to show the effects of Yerba Mate when associated with lifestyle modification. Thus, further research needs to take it into account. Finally, we surveyed subjects’ dietary intake only during the treatment period by reviewing dietary records. Therefore, dietary intake and activity levels were not accurately controlled or impartially investigated. According to the European Medicines Agency, subjects in randomized controlled trials evaluating weight control should adhere to an appropriate weight-reducing diet for a specified minimum period of time [36]. Despite these limitations, the data from our study suggested that Yerba Mate is a safe and effective anti-obesity agent.

## Conclusions

In conclusion, the results of this study revealed that Yerba Mate was a potent anti-obesity reagent that did not produce any significant adverse effects.

For full determination of the long-term effects of Yerba Mate application, a larger sample size is needed. Combined with lifestyle modifications, the effects of Yerba Mate will become more pronounced.

## Additional files

Additional file 1:
**A CONSORT checklist for the reporting of this study.** (DOC 58 kb) Additional file 2:
**Composition of test capsules provided.** (DOC 34.5 kb) Additional file 3:
**Dietary assessment parameters of the Yerba Mate and placebo groups measured at 0, 6 and 12 weeks.** (DOC 37.5 kb) Additional file 4:
**Hematology parameters of the Yerba Mate and the placebo groups measured at 0 and 12 weeks.** (DOC 56 kb) Additional file 5:
**Vital sign parameters of the Yerba Mate and the placebo groups measured at 0, 6 and 12 weeks.** (DOC 35 kb) Additional file 6:
**Side effects of the Yerba Mate and the placebo groups.** (DOC 31 kb)
