# Supplementary material for: Anti-obesity effects of Yerba Mate (Ilex Paraguariensis): a randomized, double-blind, placebo-controlled clinical trial
Source: BMC Complement Altern Med. 2015 Sep 25;15:338. doi: 10.1186/s12906-015-0859-1 (PMC4583719; doi:10.1186/s12906-015-0859-1)
Supplement: Additional file 2: — Composition of test capsules provided. (DOC 34.5 kb) [file 12906_2015_859_MOESM2_ESM.doc]

| Additional file 2 Composition of test capsules provided. | | | |
| --- | --- | --- | --- |
| Yerba Mate capsule | | Placebo capsule | |
| Component | Content (mg) | Component | Content (mg) |
| Yerba Mate powder | 333.38 mg | Corn starch | 319.2 mg |
| Corn starch | 13.12 mg | Maltodextrin | 17.5 mg |
| Magnesium Stearate | 3.5 mg | Magnesium Stearate | 3.5 mg |
|  |  | Gardenia color BA | 7 mg |
|  |  | Gardenia Blue | 1.4 mg |
|  |  | Cochineal extract color | 1.4 mg |
| Total | 350 mg | Total | 350 mg |
