# Supplementary material for: Anti-obesity effects of Yerba Mate (Ilex Paraguariensis): a randomized, double-blind, placebo-controlled clinical trial
Source: BMC Complement Altern Med. 2015 Sep 25;15:338. doi: 10.1186/s12906-015-0859-1 (PMC4583719; doi:10.1186/s12906-015-0859-1)
Supplement: Additional file 3: — Dietary assessment parameters of the Yerba Mate and placebo groups measured at 0, 6 and 12 weeks. (DOC 37.5 kb) [file 12906_2015_859_MOESM3_ESM.doc]

| Additional file 3 Dietary assessment parameters of the Yerba Mate and placebo groups measured at 0, 6 and 12 weeks. | | | | | | | | | |
| --- | --- | --- | --- | --- | --- | --- | --- | --- | --- |
|  | Yerba Mate (n=15) | | | | Placebo (n=15) | | | | *P*  value^1)^ |
|  | 0 week | 6 weeks | 12 weeks | *P* value^1)^ | 0 weeks | 6 weeks | 12 weeks | *P*  value^1)^ |
| Total calorie (kcal) | 1529.87±829.07 | 1522.10±534.20 | 1461.41±650.19 | 0.978 | 1641.15±499.08 | 1567.99±414.14 | 1926.01±573.51 | 0.029 | 0.222 |
| Carbohydrate (g) | 232.38±110.23 | 228.70±77.05 | 211.30±86.05 | 0.756 | 253.91±77.20 | 230.15±63.45^†^ | 285.95±68.91 | 0.007 | 0.083 |
| Fat (g) | 39.32±31.98 | 39.72±18.48 | 42.94±29.05 | 0.698 | 40.36±18.13 | 41.19±23.86 | 47.83±24.88 | 0.596 | 0.996 |
| Protein (g) | 63.67±36.04 | 63.66±25.04 | 58.41±25.39 | 0.833 | 60.95±19.96 | 65.48±20.78 | 75.85±24.74 | 0.096 | 0.248 |
| Fiber (g) | 17.74±8.57 | 17.44±5.35 | 15.94±4.81 | 0.678 | 20.60±6.59 | 19.19±6.44 | 24.34±6.48 | 0.026 | 0.051 |
| Values are presented as the mean ± S.D. | | | | | | | | | |
| ^1)^ Analyzed by repeated measures ANOVA. Statistically significant compared to the placebo group.  ^†^ 0 vs. 6 weeks: carbohydrate, *P*=0.029 | | | | | | | | | |
|  | | | | | | | | | |
