# Supplementary material for: Anti-obesity effects of Yerba Mate (Ilex Paraguariensis): a randomized, double-blind, placebo-controlled clinical trial
Source: BMC Complement Altern Med. 2015 Sep 25;15:338. doi: 10.1186/s12906-015-0859-1 (PMC4583719; doi:10.1186/s12906-015-0859-1)
Supplement: Additional file 4: — Hematology parameters of the Yerba Mate and the placebo groups measured at 0 and 12 weeks. (DOC 56 kb) [file 12906_2015_859_MOESM4_ESM.doc]

| Additional file 4 Hematology parameters of the Yerba Mate and the placebo groups measured at 0 and 12 weeks. | | | | | | | |
| --- | --- | --- | --- | --- | --- | --- | --- |
|  | Yerba Mate (n=15) | | | Placebo (n=15) | | | *P* value^2)^ |
|  | 0 weeks | 12 weeks | *P* value^1)^ | 0 weeks | 12 weeks | *P* value^1)^ |
| WBC (10³/㎕) | 6.5±1.7 | 6.4±1.4 | 0.814 | 5.8±1.5 | 5.8±1.2 | 0.841 | 0.925 |
| RBC (100³/㎕) | 4.5±0.3 | 4.6±0.3 | 0.375 | 4.4±0.3 | 4.6±0.4 | <.0001 | 0.045 |
| Hemoglobin (g/dL) | 13.8±1.4 | 13.7±1.4 | 0.619 | 13.5±1.1 | 13.8±1.3 | 0.004 | 0.020 |
| Hematocrit (%) | 40.3±3.4 | 40.7±3.4 | 0.476 | 40.1±2.9 | 41.1±3.1 | <.0001 | 0.031 |
| PLT (%) | 275.5±61.7 | 282.1±84.4 | 0.746 | 267.5±43.9 | 268.3±61.8 | 0.590 | 0.524 |
| SEG (%) | 57.9±9.5 | 56.0±7.8 | 0.257 | 55.2±7.3 | 55.0±7.4 | 0.754 | 0.552 |
| Lymphocyte (%) | 32.1±8.4 | 33.6±7.7 | 0.403 | 36.8±6.5 | 34.7±6.0 | 0.298 | 0.188 |
| Monocyte (%) | 6.2±1.7 | 6.5±1.6 | 0.604 | 5.5±1.4 | 6.7±3.1 | 0.111 | 0.360 |
| Eosinophil (%) | 3.4±2.5 | 3.3±2.2 | 0.882 | 2.1±1.1 | 3.2±2.5 | 0.049 | 0.097 |
| Basophil (%) | 0.3±0.1 | 0.5±0.3 | 0.008 | 0.3±0.2 | 0.4±0.2 | 0.076 | 0.294 |
| ALP (IU/L) | 65.1±14.8 | 62.5±14.9 | 0.133 | 62.6±17.2 | 60.0±16.9 | 0.221 | 0.855 |
| γ-GT (IU/L) | 25.1±13.6 | 28.1±16.9 | 0.334 | 22.9±17.4 | 21.7±17.1 | 0.510 | 0.486 |
| AST (IU/L) | 20.9±7.3 | 21.6±4.9 | 0.416 | 20.7±5.2 | 22.3±4.7 | 0.282 | 0.653 |
| ALT (IU/L) | 22.2±10.8 | 24.3±11.6 | 0.342 | 19.5±7.3 | 24.3±8.5 | 0.045 | 0.620 |
| Glucose (mg/dL) | 87.9±15.5 | 87.7±9.8 | 0.929 | 88.3±8.1 | 88.9±12.7 | 0.596 | 0.645 |
| Bilirubin (mg/dL) | 0.8±0.3 | 0.8±0.4 | 0.759 | 0.8±0.3 | 0.8±0.5 | 0.206 | 0.437 |
| Protein (g/dL) | 7.2±0.3 | 7.5±0.4 | 0.007 | 7.2±0.3 | 7.5±0.2 | 0.001 | 0.812 |
| Albumin (g/dL) | 4.4±0.2 | 4.5±0.2 | 0.045 | 4.4±0.2 | 4.5±0.2 | 0.001 | 0.890 |
| Blood Urea Nitrogen (mg/dL) | 12.0±2.7 | 13.9±3.1 | 0.069 | 13.2±4.2 | 14.1±3.0 | 0.048 | 0.747 |
| Creatinine (mg/dL) | 0.7±0.2 | 0.6±0.1 | 0.076 | 0.6±0.1 | 0.6±0.1 | 0.261 | 0.163 |
| Specific Gravity | 1.0±0.0 | 1.0±0.0 | 0.779 | 1.0±0.0 | 1.0±0.0 | 0.133 | 0.228 |
| pH | 6.1±0.8 | 6.0±1.0 | 0.793 | 6.0±0.8 | 6.0±0.9 | 0.936 | 0.810 |
| Values are presented as the mean ± S.D. | | | | | | | |
| ^1)^ Analyzed by paired t test. Statistically significant compared to the baseline. | | | | | | | |
| ^2)^ Analyzed by repeated measures ANOVA. Statistically significant compared to the placebo group. | | | | | | | |
