# Supplementary material for: Anti-obesity effects of Yerba Mate (Ilex Paraguariensis): a randomized, double-blind, placebo-controlled clinical trial
Source: BMC Complement Altern Med. 2015 Sep 25;15:338. doi: 10.1186/s12906-015-0859-1 (PMC4583719; doi:10.1186/s12906-015-0859-1)
Supplement: Additional file 5: — Vital sign parameters of the Yerba Mate and the placebo groups measured at 0, 6 and 12 weeks. (DOC 35 kb) [file 12906_2015_859_MOESM5_ESM.doc]

| Additional file 5 Vital sign parameters of the Yerba Mate and the placebo groups measured at 0, 6 and 12 weeks. | | | | | | | | | |
| --- | --- | --- | --- | --- | --- | --- | --- | --- | --- |
|  | Yerba Mate (n=15) | | | | Placebo (n=15) | | | | *P* value^1)^ |
|  | 0 week | 6 weeks | 12 weeks | *P* value^1)^ | 0 week | 6 weeks | 12 weeks | *P* value^1)^ |
| Systolic pressure (mmHg) | 116.0±11.0 | 118.6±8.9 | 121.1±7.8 | 0.166 | 118.1±11.9 | 122.5±14.2 | 120.1±13.5 | 0.398 | 0.255 |
| Diastolic pressure (mmHg) | 73.5±8.2 | 77.3±6.7 | 76.6±6.0 | 0.067 | 74.5±9.6 | 78.5±12.9 | 77.8±11.2 | 0.229 | 0.953 |
| Pulse (mmHg) | 75.3±9.1 | 69.9±7.7 | 72.3±7.6 | 0.048 | 74.0±5.6 | 74.6±6.7 | 77.3±8.9 | 0.327 | 0.087 |
| Body temperature (℃) | 36.4±0.4 | 36.4±0.3 | 36.3±0.3 | 0.485 | 36.5±0.2 | 36.5±0.3 | 36.5±0.3 | 0.921 | 0.606 |
| Values are presented as the mean ± S.D.  ^1)^ Analyzed by repeated measures ANOVA. Statistically significant compared to the placebo group. | | | | | | | | | |
