# Supplementary material for: Anti-obesity effects of Yerba Mate (Ilex Paraguariensis): a randomized, double-blind, placebo-controlled clinical trial
Source: BMC Complement Altern Med. 2015 Sep 25;15:338. doi: 10.1186/s12906-015-0859-1 (PMC4583719; doi:10.1186/s12906-015-0859-1)
Supplement: Additional file 6: — Side effects of the Yerba Mate and the placebo groups. (DOC 31 kb) [file 12906_2015_859_MOESM6_ESM.doc]

|  | Additional file 6 Side effects of the Yerba Mate and the placebo groups. | | | | | |
| --- | --- | --- | --- | --- | --- | --- |
|  |  | Yerba Mate (n=15) | | Placebo (n=15) | *P* value^2)^ | |
|  | Side effects (Yes/No) | 6/9^1)^ | | 7/8 | 0.713 | |
| *^1)^ N* | | |  | | | |
| ^2)^ Analyzed by chi-square test (Fisher's exact test). | | | | | |  |
